# Obesity is associated with early recurrence on breast cancer patients that achieved pathological complete response to neoadjuvant chemotherapy

**DOI:** 10.1038/s41598-022-25043-2

**Published:** 2022-12-07

**Authors:** Francisco Acevedo, Benjamín Walbaum, Sabrina Muñiz, Militza Petric, Raúl Martínez, Constanza Guerra, Marisel Navarro, Miguel Córdova-Delgado, Mauricio P. Pinto, Cesar Sánchez

**Affiliations:** 1 Department of Hematology and Oncology, School of Medicine, Pontificia Universidad Católica de Chile, Diagonal Paraguay 362, 6th FL. Rm 608, 8330077 Santiago, Chile; 2 Division of Oncological Surgery, Dr. Sotero del Rio Hospital and Healthcare Complex, Santiago, Chile

**Keywords:** Cancer, Breast cancer

## Abstract

Pathological complete response (pCR) after neoadjuvant chemotherapy (NCT) is associated with good long-term prognosis in breast cancer (BC) patients. However, some patients still recur and eventually die from this disease. For years, clinical stage at diagnosis has been consistently linked to recurrence and survival in the pCR setting. Herein, we aimed to identify other potential predictors of recurrence and survival in patients that achieved pCR. We performed a retrospective analysis of patients diagnosed between 2011 and 2020 in our center. We calculated overall survival (OS), invasive disease-free survival (IDFS), distant disease-free survival (DDFS), and BC-specific survival (BCSS). Among the 241 patients included into our study 36% were obese (Body Mass Index (BMI) > 29.9 kg/m^2^) and 47% were stage III. Multivariate analysis confirmed that obesity was a significant risk factor associated with early recurrence and poorer survival in these patients. In summary, obesity and clinical stage predict early recurrence and poorer survival in patients that achieved pCR after NCT. Pending further investigation and based on our findings we speculate that weight management could be beneficial for this subset of patients. To our knowledge, this is the first Latin American report linking obesity and recurrence within this setting.

## Introduction

Data from high income countries indicates that > 90% of newly diagnosed breast cancer (BC) cases correspond to non-metastatic disease^1–3^. Systemic therapy (chemotherapy, endocrine therapy or humanized monoclonal antibodies) in these patients aims to improve clinical outcomes by controlling micro-metastatic foci and loco regional disease, decreasing distant/local relapse^4^. In recent decades. the preoperative use of systemic therapy with curative intent (i.e. neoadjuvant chemotherapy (NCT)) has become the standard of care for locally advanced BC and early stage human epidermal growth factor receptor-type 2 positive (HER2+) or triple negative BC (TNBC)^5–7^.

Although several reports demonstrate that patients that achieve a pathological complete response (pCR) following NCT display favorable prognoses, a proportion of them still can experience disease recurrence and eventually die of BC^8–10^. In this regard, a variety of factors have been proposed to determine prognosis on patients that achieve pCR. However, studies confirm the prognosis in this subset of patients is consistently associated with tumor burden measures such as tumor size, lymph node compromise and/or clinical stage (cTNM)^11–13^.

On the other hand, previous studies have demonstrated that obesity is associated with larger, higher grade tumors^14,15^, increased risks of recurrence and death^16,17^, and thereby poorer survival^18,19^ among BC patients. Herein we identified potential predictors of recurrence and survival in a retrospective cohort of non-metastatic patients that achieved pCR following NCT.

## Methods

### Patients and ethics approval

Registry-based, retrospective real-world analysis of Chilean BC patients, including patients from public (South-Eastern Metropolitan Health Service (SSMSO)), and private (UC-Christus Health Network) hospitals in Santiago; the former a public hospital (PH) and the latter a university hospital (UH).

We included stages I to III BC patients treated with NCT from January 2011 through December 2020. We excluded metastatic patients at diagnosis, those without pathological response information in surgical biopsy, and patients who received NCT as part of their recurrence. All methods were carried out in accordance with relevant guidelines and regulations. All experimental protocols were approved by the Scientific and Ethics committee for Health Sciences; Protocol ID# 200115017, dated on August 6th, 2020, and updated on December 2nd, 2021. Collected information of patients did not include sensitive data. Therefore, the abovementioned ethics committee granted a waiver of consent for the retrospective inclusion of patients into this study.

### Data collection and categorization

We extracted clinical, pathological and follow-up information from the electronic medical record.

Variables evaluated were year and age of diagnosis, cancer family history, hospital of origin (PH vs UH), health insurance system (public vs private), cTNM stage^20^, body mass index (BMI: weight/height^2^) measured at the beginning of treatment, comorbidities, regimen of NCT and radiotherapy use. Survival data were extracted first from medical records, otherwise they were obtained from the Chilean Civil Registry (https://www.registrocivil.cl). Standard immunohistochemistry (IHC) was performed to determine hormone receptor (HR) and HER2 tumor status following American Society of Clinical Oncology/College of American Pathologists (ASCO/CAP) guidelines^21^. The cut-off value for ER and/or PR positivity was > 1% and HER2 was considered positive if IHC was 3 plus or FISH positive if 2 plus. In this way, we were able to categorize patients in four groups: HR+/HER2−, HR+/HER2+, HR−/HER2+ (equivalent to HER2-enriched) and HR−/HER2− (also called TNBC).

### Pathological complete response

pCR was defined as the absence of residual invasive disease in the breast and in the axillary lymph nodes (ypT0/is N0) at the completion of the NCT^8^.

### Statistics

Descriptive statistics were used to describe baseline patient characteristics. Chi-square/Kruskal–Wallis tests were used for categorical/continuous variables. Invasive Disease-Free Survival (IDFS), Distant Disease-Free Survival (DDFS), BC Specific Survival (BCSS) and Overall Survival (OS) were measured from the time of diagnosis to the event or lost to follow-up. We performed a univariate Cox regression analysis for each factor in order to identify those associated with prognosis. Characteristics with a p-value < 0.2 and those with less than 10% of missing data were considered for inclusion in the Cox regression multivariate analysis. By the missing data criterion (10%), the variables “comorbidities” and “use of metformin” were excluded from the multivariate analyses, also, the “lymph node positive” variable was excluded due to its dependency on stage III (the latter was included). For testing normality of continuous data, we used Shapiro–Wilk's method. For all multivariate cox models presented, the assumptions of this approach were tested, where the proportional hazards assumption was checked using statistical tests and graphical diagnostics based on the scaled Schoenfeld residuals. Survival curves were presented using Kaplan–Meier methods and groups were compared using log-rank. A p < 0.05 was considered to define statistical significance. We use STATA v.15.1 and R v.4.1.0 including “knitr”, “finalfit”, “survival”, “survminer” and “dplyr” packages for all analyses.

## Results

Our study included a total of 5,191 BC patients diagnosed between 2011 and 2020. As expected, the majority (n = 4791; 92.3%) were non-metastatic (stage I–III) cases. Among these, 957 (20.0%) received NCT. Within this subset, we retrieved medical records and pCR information from 882 patients (92.2%); 241 (27.3%) achieved pCR (Supplementary Fig. S1). Basic patient characteristics are summarized in Table 1. Briefly, median age at diagnosis was 50.27 year-old (range 24.46–78.84), median BMI was 27.82 kg/m^2^ (range 18.51–50.15) and > 70% of patients were either overweight or obese. Most patients (60.2%) were HR− and the most frequent BC subset was HR−/HER2+.

**Table 1:** Patients’ basic characteristics.

Median values; units	Total n = 241
Age, years (range)	50.27 (24.46–78.84)
BMI, kg/m^2^ (range)	27.82 (18.51–50.15)

Variables	n (%)
< 40 yr-old patients	45 (18.7)
**Hospital type**	
Public	96 (39.8)
Private	145 (60.2)
**Type of health insurance**	
Public	179 (75.2)
Private	59 (24.8)
**BMI category**	
Normal (< 25.0 kg/m^2^)	64 (29.0)
Overweight (25.0–29.99 kg/m^2^)	79 (35.7)
Obese (> 29.9 kg/m^2^)	78 (35.3)
**Stage at diagnosis**	
Stage I or II	124 (52.5)
Stage III	112 (47.5)
**BC subset**	
HR+/HER2−	39 (16.2)
HR+/HER2+	57 (23.7)
HR−/HER2+	84 (34.9)
HR−/HER2−	61 (25.3)
+ Lymph nodes	155 (67.7)
**Type of chemotherapy**	
Anthracycline + Taxane	222 (92.5)
Anthracycline only	3 (1.2)
Taxane only	15 (6.2)
With children^a^	184 (85.2)
+ Family history^a^	151 (71.2)
+ Ovarian cancer^a^	84 (39.8)
+ Comorbidities^a^	93 (47.4)
+ Hypertension^a^	38 (18.7)
+ Type-2 diabetes^a^	15 (7.5)
+ Hypothyroidism^a^	22 (11.9)
Use of metformin^a^	22 (12.4)
Use of statins^a^	13 (7.5)
Use of aspirin^a^	12 (6.9)
+ Adjuvant hormone therapy^a^	61 (34.7)
+ Adjuvant radiotherapy^a^	135 (91.8)

*yr* year, *BMI* body mass index, *BC* breast cancer, *HR* hormone receptor, *HER2* human epidermal growth factor type-2 receptor.

^a^< 10% of missing data.

**Figure 1:**
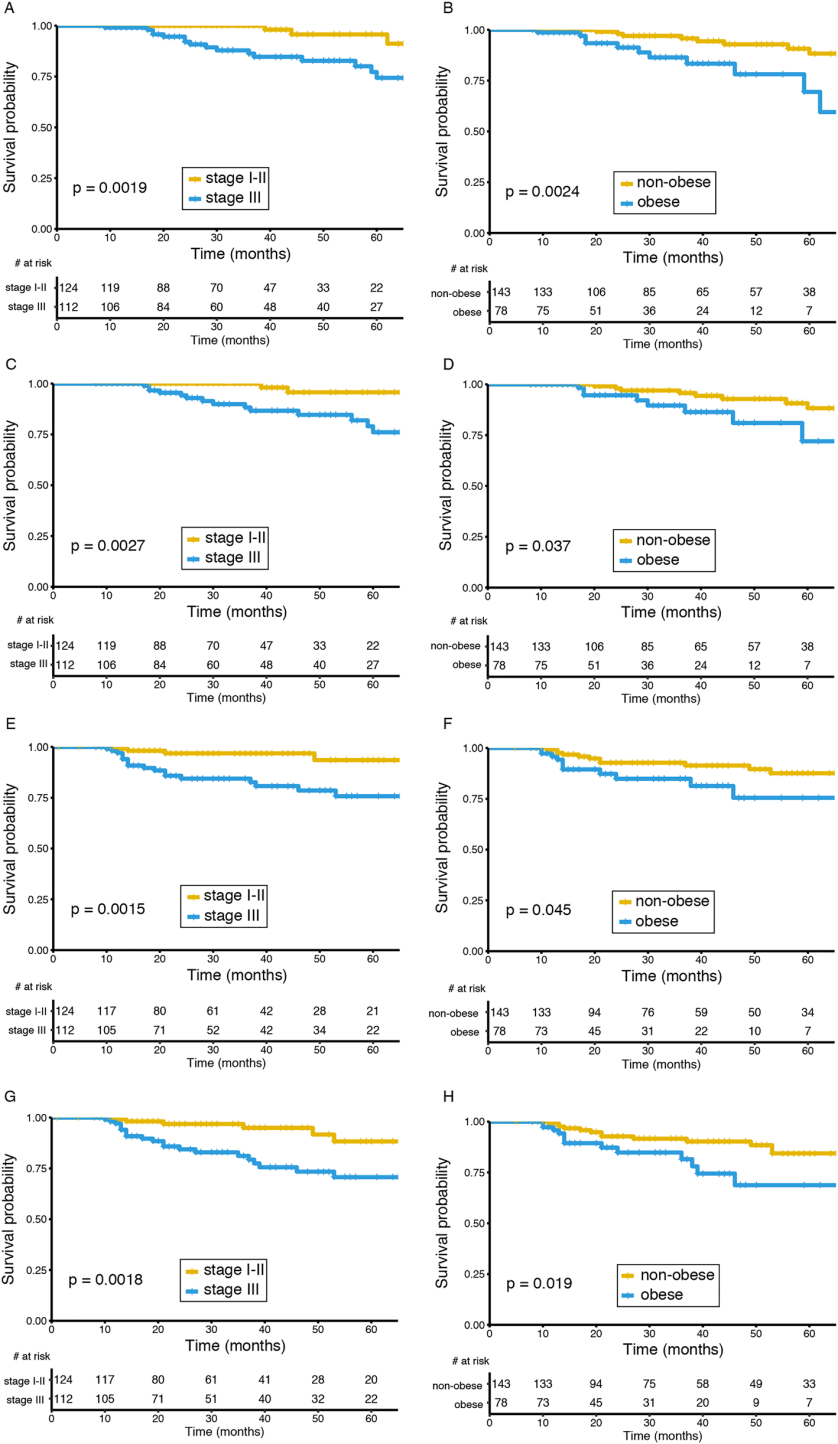
Stage at diagnosis and BMI status affect survival rates in patients that achieved pathological complete response after neoadjuvant chemotherapy. (**A**) Overall survival (OS) curves comparing stage I/II versus stage III (LogRank p = 0.0019). (**B**) OS curves comparing non-obese versus obese (LogRank p = 0.0024). (**C**) Breast cancer specific survival (BCSS) curves comparing stage I/II versus stage III (LogRank p = 0.0027). (**D**) BCSS curves comparing non-obese versus obese (LogRank p = 0.037). E. Distant disease-free survival (DDFS) curves comparing stage I/II versus stage III (LogRank p = 0.0015). (**F**) DDFS curves comparing non-obese versus obese (LogRank p = 0.045). (**G**) Invasive-disease free survival (IDFS) curves comparing stage I/II versus stage III (LogRank p = 0.0018). (**H**) IDFS curves comparing non-obese versus obese (LogRank p = 0.019).

Initially, we sought to determine which variables had a significant impact on patient overall survival (OS) and performed a univariate analysis that included both demographic and clinical variables (Supplementary Table 1). Thus, when we categorized patients as obese or not obese (using a cutoff of BMI 29.9 kg/m^2^) we found a significant association with OS (p = 0.005) and BCSS (p = 0.045). In addition, age (p = 0.019) and stage III at diagnosis (p = 0.006) were also significantly associated with poorer OS. Next, we selected those variables that were significantly associated with OS and built a multivariate model to confirm these findings (Table 2). We also evaluated the impact of stage III and obesity on different survival measures including OS, BCSS, DDFS and IDFS using the Kaplan–Meier method. Figure 1 summarizes these results and show survival curves comparing stage I/II versus stage III and non-obese versus obese on OS (Fig. 1A,B), BCSS (Fig. 1C,D), DDFS (Fig. 1E,F) and IDFS (Fig. 1G,H). Please note that in all cases stage III at diagnosis or obesity significantly decrease patient survival.

**Table 2:** Multivariate analyses of variables associated with survival.

Variable	Univariate			Multivariate		
	HR	[CI: 95%]	p-value	HR	[CI: 95%]	p-value
Age at diagnosis	1.04	[1.01–1.08]	0.019*	1.04	[1.00–1.08]	0.041*
**Hospital type**						
Public	Ref	–	–	–	–	–
Private	2.16	[0.72–6.47]	0.17	1.18	[0.38–3.71]	0.77
**Obesity**						
(−)	Ref	–	–	–	–	–
( +)	3.81	[1.51–9.59]	0.005*	3.90	[1.53–9.98]	0.004*
**Stage at diagnosis**						
I or II	Ref	–	–	–	–	–
III	5.62	[1.65–19.21]	0.006*	5.00	[1.45–17.21]	0.011*

* indicates p < 0.05

**Figure 2:**
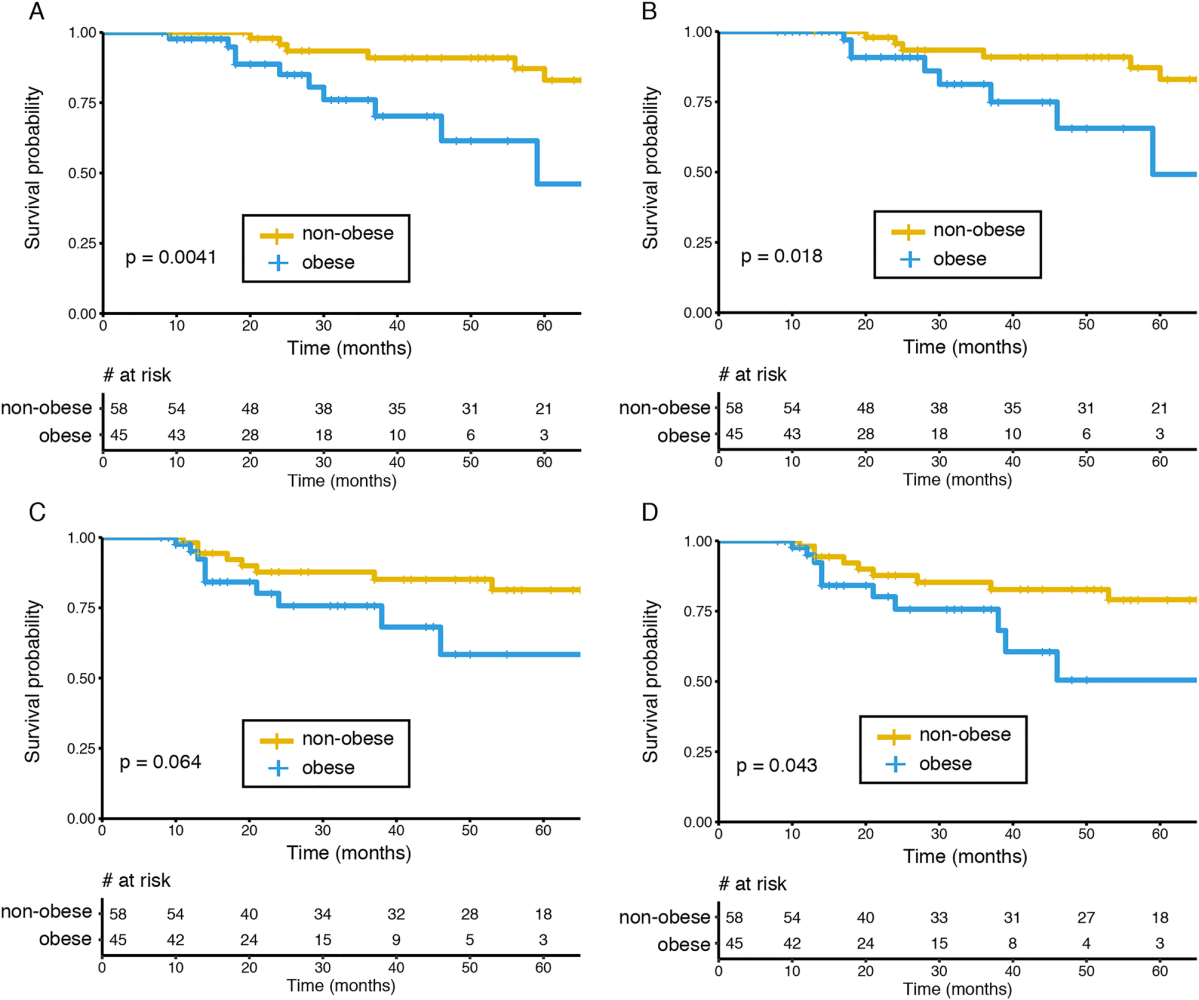
Obesity is associated with poorer survival in stage III patients. (**A**) OS curves comparing non-obese versus obese (LogRank p = 0.0041). (**B**) BCSS curves comparing non-obese versus obese (LogRank p = 0.018). (**C**) DDFS curves comparing non-obese versus obese (LogRank p = 0.064). (**D**) IDFS curves comparing non-obese versus obese (LogRank p = 0.043).

Interestingly, subsequent analyses demonstrated a codependence between stage III and obesity and a significant relation between these variables in our cohort (Supplementary Table S2). Indeed, obese patients were diagnosed in stage III in a higher proportion versus stages I or II (57.7% vs. 41.7%, p = 0.024). In contrast, no relationship was found between stage and age. Therefore, based on this result we performed additional subgroup analyses and this time we analyzed the impact of obesity on stage I/II or stage III cases separately. Table 3 shows that age at diagnosis had no significant effect on survival on stage I/II, but a marginal significance (p < 0.1) for stage III cases. We also confirmed a significant effect of obesity upon stage III cases but not on stage I/II. As described above, in order to further confirm our findings, we evaluated the impact of obesity on survival, however this time we only included stage III cases. Figure 2 confirms a significant effect of obesity on OS (Fig. 2A), BCSS (Fig. 2B) and IDFS (Fig. 2D), and a marginal significance for DDFS (Fig. 2C; p = 0.064) using the Kaplan–Meier method. Lastly, 11.6% of patients (n = 28) in our cohort recurred; the main features of these recurrence events are summarized in supplementary Table 3.

**Table 3:** Univariate and multivariate analyses of variables associated with survival by stage at diagnosis.

Variable	Univariate			Multivariate		
	HR	[CI: 95%]	p-value	HR	[CI: 95%]	p-value
**Stages I or II cases**						
Age at diagnosis	1.07	[0.96–1.18]	0.211	1.07	[0.97–1.19]	0.194
**Obesity**						
(−)	Ref	–	–	–	–	–
( +)	1.43	[0.13–15.96]	0.773	1.67	[0.14–19.55]	0.681
**Stage III cases**						
Age at diagnosis	1.03	[1.00–1.07]	0.067	1.04	[1.00–1.08]	0.080
**Obesity**						
(−)	Ref	–	–	–	–	–
( +)	4.16	[1.46–11.83]	0.008*	4.31	[1.51–12.27]	0.006*

* indicates p < 0.05

## Discussion

Several studies confirm BMI is a key prognostic factor in BC^22–24^. It is well documented that obesity increases the risk of recurrence and death by 35–40% in estrogen receptor positive BCs^25^. Unfortunately, obesity rates in Latin America have skyrocketed in recent decades becoming a public health concern. Chile is no exception to this problem. In fact, according to the organization for economic cooperation and development (OECD)^26^ Chile currently ranks second in obesity rates among OECD members. To our knowledge, this is the first Latin American report linking obesity, early recurrence, and prognosis in the subset of BC patients that achieved pCR after NCT. While previous reports demonstrate that obesity is not a relevant risk factor for BC in premenopausal women, it is associated to poorer overall outcomes in BC patients, regardless of their menopause status^25^. The effect of obesity on patient prognosis could be attributed to several factors including biological aspects specifically linked to obesity such as increased estrogen production or increases in inflammatory mediators. However, this could also be explained by medical decisions including reductions in chemotherapy dose or dose capping, aiming to avoid treatment-related toxicities on patients. Usually, obese patients display larger body surface areas (BSA) that reduce the effect of systemic therapies. In this regard, a recent update in ASCO guidelines recommends maintaining a full dose particularly on obese patients^27^.

In line with previous reports, our results confirm that initial tumor load (cTNM) is the main clinical factor associated with pCR^11^. A handful of previous studies have sought to discover other prognostic factors in BC patients that achieve pCR after NCT^11–13^. However, tumor burden measures at diagnosis (i.e. tumor size, lymph node compromise, clinical stage (cTNM)) are the only ones that have been consistently associated with patient outcomes in the literature^9,11,13^. A study by Asaoka et al. found that advanced stage status or HER2-positivity were significant predictors of patient survival on multivariate analyses^12^. Unfortunately, we did not find a significant association between BC subtype and prognosis on patients that achieved pCR in our study. We speculate this could be attributed to a small sample size, the length of clinical follow up and environmental factors or genetic characteristics of our population.

As expected, survival rates in our cohort surpass the average for BC patients with a 3-year OS of 93.2%. This is obviously due to a patient selection bias in our study that included only those that achieved a pCR. Still, 11.6% of patients recurred and 8% died by BC; these numbers are lower compared to similar studies. Differences could be attributed to several factors. First, 47.5% of patients in our cohort were advanced stage cases (stage III) versus 27.7%^28^ or 32%^13^ of stage III reported by similar studies. Secondly, 35.3% of patients in our cohort were obese, this is > 2-fold the value reported by similar studies that indicate14%^11^ or 16%^29^. Interestingly, a study by O’Shaughnessy et al. reports similar rates of obesity with 37% in a cohort of 217 patients from the US Oncology Network^28^. However, this study was limited to HER2 + BC cases and investigators did not find an association between obesity and recurrence. Intriguingly, our study also found a significant association between obesity and stage III cases. The association between BMI and BC stage has been previously reported by others^30^. In this study, investigators hypothesized that the increase in breast size in obese women causes a delay in tumor discovery and diagnosis. Despite this, our analyses confirmed the impact of obesity on patient survival even within the subset of stage III cases (Fig. 2). Evidently, the association between obesity and prognosis in our cohort might also be affected by ethnicity. The Chilean population is a complex mixture of Amerindians and XVI/XVII century Spanish settlers. Another fraction of this population (± 4%) is composed by XIX century immigrants that included Germans, Croatians, Arabs, and Italians^31^. Interestingly, recent studies have postulated that ancestry plays a role on the adverse effects of obesity on BC risk and survival^32^. Future studies should further confirm and validate these findings and determine the mechanism and the potential causality relationship in this association. Lastly, and in line with the abovementioned studies, Latin American reports consistently confirm a higher proportion of late stage cases in the region compared to more developed countries^33,34^. We speculate that limited resources and poorer access to healthcare in the region translate into a larger proportion of women remaining undiagnosed for longer time periods and/or delaying the initiation of their treatment after diagnosed, causing an increase in late-stage diagnosis and poorer outcomes.

Overall, based on our findings we speculate that the implementation of weight control or weight loss programs could be of benefit for obese patients undergoing NCT, however this must be further assessed and validated on large clinical trials, specifically designed for this purpose. Reports suggest that fasting-mimetic diets, such as calorie restriction diets improve both physical performance and chemotherapy efficacy on BC patients, however the evidence is still weak^35^. Regarding a potential mechanism, investigators speculate that fasting normal cells enter mitotic arrest or a quiescent state, and therefore become less sensitive to chemotherapeutic drugs. In contrast, tumor cells remain mitotically active thereby maintaining their sensitivity to chemotherapy, increasing therapeutic efficacy^36^.

Interestingly, our data indicate that the brain was the most frequent distant site of metastasis in our cohort (n = 13; 5.4%; Supplementary Table 3). Previous reports have described an association between higher stage at diagnosis and brain metastases (BM)^37^. Similarly, HR negativity or HER2+ status have been associated with BM following breast-conserving therapy^38^. Unfortunately, we did not find such association in our cohort. Even though the literature in this topic is scarce studies have demonstrated that obesity affects the neurovascular unit causing disruptions on the blood brain barrier^39^ that may explain the observed prevalence of BM in our cohort. Again, future prospective studies should explore this potential mechanism.

Our study has several limitations, most of them related to the retrospective nature of the study, including short follow-up, which may account for early recurrence events, which also probably translates into missing of late events, something often seen in hormone-sensitive tumors. Another limitation was the relatively low number of cases, compared to similar studies, this made unfeasible a reliable comparison of risk among BC subtypes. It is noteworthy that the most frequent BC subset in our cohort was HR−/HER2+. Thus, since our study covered an extensive period (2011–2020) many changes in clinical practice and particularly on NCT regimes and systemic treatments may have occurred over the last decade that could impact patient outcomes, however this is unlikely to modify our general conclusions. Similarly, given that most obese patients in our cohort were stage III we cannot rule out a potential effect of treatment heterogeneity on recurrence and survival.

In summary, our study demonstrates that BC patient prognosis and recurrence among those that achieved pCR after NCT is negatively affected by advanced stage at diagnosis (stage III) and by obesity. To our knowledge, our study is the first of its kind in Latin America, providing real world evidence in this topic.

## Supplementary Information

Supplementary Information.

## Data Availability

All datasets generated and analyzed during the current study are included into the manuscript or in supplementary files; further inquiries can be directed to the corresponding author on reasonable request.
