# Supplementary material for: Obesity is associated with early recurrence on breast cancer patients that achieved pathological complete response to neoadjuvant chemotherapy
Source: Sci Rep. 2022 Dec 7;12:21145. doi: 10.1038/s41598-022-25043-2 (PMC9729290; doi:10.1038/s41598-022-25043-2)
Supplement: Supplementary file 1 — Supplementary Information. [file 41598_2022_25043_MOESM1_ESM.docx]

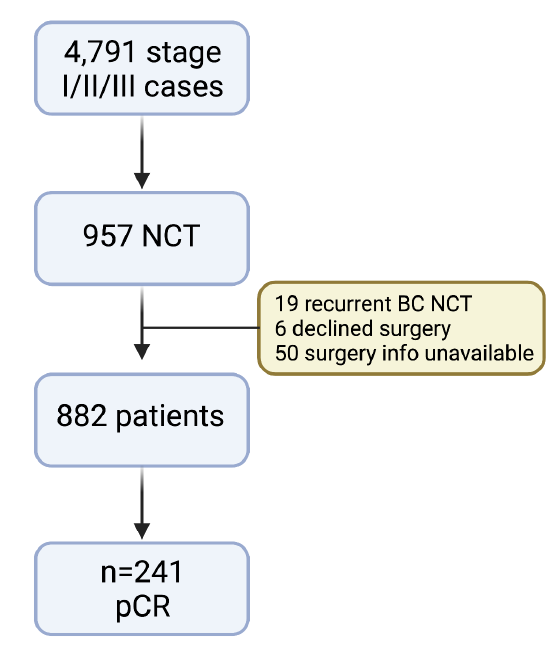


Supplementary Figure 1. Patients included in the current report. Abbreviations: NCT: neoadjuvant chemotherapy; BC: breast cancer; pCR: pathological complete response

Supplementary Table 1. Univariate analysis of 241 BC patients with pCR.

|  |  |  | **OS** |  | **BSC** |  | **IDFS** |  | **DDFS** |  |
| --- | --- | --- | --- | --- | --- | --- | --- | --- | --- | --- |
| **Variable** |  | **N or mean** | **HR [CI: 95%]** | **p-value** | **HR [CI: 95%]** | **p-value** | **HR [CI: 95%]** | **p-value** | **HR [CI: 95%]** | **p-value** |
| Age | Continuous | 51.4 | 1.04 [1.01-1.08] | 0.019 | 1.03 [0.99-1.07] | 0.144 | 1.02 [0.99-1.05] | 0.15 | 1.01 [0.98-1.05] | 0.408 |
| Hospital type | *Public* | 96 | Ref. | 0.17 | Ref. | 0.201 | Ref. | 0.747 | Ref. | 0.545 |
|  | *Private* | 145 | 2.16 [0.72-6.47] |  | 2.27 [0.65-8.01] |  | 1.15 [0.50-2.63] |  | 1.34 [0.52-3.43] |  |
| Obesity | (-) | 143 | Ref. | 0.005 | Ref. | 0.045 | Ref. | 0.024 | Ref. | 0.051 |
|  | (+) | 78 | 3.81 [1.51-9.59] |  | 2.75 [1.02-7.42] |  | 2.46 [1.13-5.35] |  | 2.32 [1.00-5.40] |  |
| Stage at diagnosis | I or II | 124 | Ref. | 0.006 | Ref. | 0.01 | Ref. | 0.004 | Ref. | 0.004 |
|  | III | 112 | 5.62 [1.65-19.21] |  | 7.01 [1.59-30.90] |  | 3.82 [1.54-9.47] |  | 4.87 [1.65-14.40] |  |
| BC subset | *HR+/HER2-* | 39 | Ref. | 0.835 | Ref. | 0.639 | Ref. | 0.373 | Ref. | 0.382 |
|  | *HR+/HER2+* | 57 | 0.89 [0.18-4.41] |  | 0.86 [0.12-6.13] |  | 0.40 [0.10-1.59] |  | 0.31 [0.06-1.59] |  |
|  | *HR-/HER2+* | 84 | 1.51 [0.40-5.71] |  | 1.98 [0.41-9.52] |  | 1.06 [0.40-2.84] |  | 0.94 [0.31-2.80] |  |
|  | *HR-/HER2-* | 61 | 1.34 [0.33-5.36] |  | 1.70 [0.33-8.80] |  | 0.72 [0.23-2.25] |  | 0.89 [0.27-2.93] |  |
| Lymph nodes | (-) | 74 | Ref. | 0.066 | Ref. | 0.111 | Ref. | 0.062 | Ref. | 0.039 |
|  | (+) | 155 | 6.61 [0.88-49.64] |  | 5.21 [0.68-39.72] |  | 3.15 [0.94-10.50] |  | 8.29 [1.11-61.87] |  |
| Type of chemotherapy | *Anthracycline+Taxane* | 222 | Ref. | 0.817 | Ref. | 0.603 | Ref. | 0.516 | Ref. | 0.762 |
|  | *Anthracycline only* | 3 | 1.96 [0.26-14.83] |  | N.A |  | N.A |  | N.A |  |
|  | *Taxane only* | 15 | 1.31 [0.17-9.91] |  | 1.64 [0.21-12.50] |  | 1.95 [0.46-8.32] |  | 1.05 [0.14-7.87] |  |
| Family history | (-) | 61 | Ref. | 0.845 | Ref. | 0.863 | Ref. | 0.321 | Ref. | 0.313 |
|  | (+) | 151 | 1.11 [0.40-3.07] |  | 1.11 [0.35-3.47] |  | 1.64 [0.62-4.38] |  | 1.75 [0.59-5.21] |  |
| Comorbidities | (-) | 103 | Ref. | 0.014 | Ref. | 0.095 | Ref. | 0.078 | Ref. | 0.184 |
|  | (+) | 93 | 4.08 [1.32-12.56] |  | 2.73 [0.84-8.92] |  | 2.19 [0.92-5.22] |  | 1.90 [0.74-4.91] |  |
| Hypertension | (-) | 165 | Ref. | 0.007 | Ref. | 0.055 | Ref. | 0.601 | Ref. | 0.38 |
|  | (+) | 38 | 3.97 [1.45-10.85] |  | 3.19 [0.97-10.41] |  | 1.34 [0.45-3.97] |  | 1.65 [0.54-5.04] |  |
| Hypothyroidism | (-) | 163 | Ref. | 0.029 | Ref. | 0.016 | Ref. | 0.003 | Ref. | 0.034 |
|  | (+) | 22 | 3.68 [1.14-11.87] |  | 4.38 [1.31-14.60] |  | 3.93 [1.59-9.72] |  | 3.06 [1.09-8.61] |  |
| Type-2 diabetes | (-) | 184 | Ref. | 0.002 | Ref. | 0.043 | Ref. | 0.169 | Ref. | 0.091 |
|  | (+) | 15 | 5.34 [1.88-15.16] |  | 3.80 [1.04-13.84] |  | 2.35 [0.70-7.97] |  | 2.92 [0.84-10.08] |  |
| Use of metformin | (-) | 156 | Ref. | 0.095 | Ref. | 0.738 | Ref. | 0.819 | Ref. | 0.462 |
|  | (+) | 22 | 2.60 [0.85-7.98] |  | 0.71 [0.09-5.44] |  | 0.84 [0.20-3.63] |  | 0.47 [0.06-3.53] |  |
| Use of statins | (-) | 161 | Ref. | 0.276 | Ref. | 0.791 | Ref. | 0.883 | Ref. | 0.972 |
|  | (+) | 13 | 2.29 [0.52-10.13] |  | 1.32 [0.17-10.19] |  | 0.86 [0.12-6.42] |  | 1.04 [0.14-7.85] |  |
| Use of aspirin | (-) | 162 | Ref. | 0.235 | Ref. | 0.499 | Ref. | 0.809 | Ref. | 0.563 |
|  | (+) | 12 | 2.15 [0.61-7.58] |  | 1.68 [0.37-7.64] |  | 1.20 [0.28-5.14] |  | 1.55 [0.35-6.76] |  |
| Adjuvant hormone therapy | (-) | 115 | Ref. | 0.342 | Ref. | 0.286 | Ref. | 0.844 | Ref. | 0.999 |
|  | (+) | 61 | 0.53 [0.15-1.95] |  | 0.43 [0.09-2.03] |  | 0.91 [0.36-2.29] |  | 1.00 [0.36-2.76] |  |
| Adjuvant radiotherapy | (-) | 12 | Ref. | 0.366 | Ref. | 0.898 | Ref. | 0.606 | Ref. | 0.725 |
|  | (+) | 135 | 0.50 [0.11-2.27] |  | 0.87 [0.11-6.91] |  | 1.70 [0.23-12.73] |  | 1.44 [0.19-10.89] |  |

Supplementary Table 2. Relation between Obesity status and age/stage

|  | Non-Obese (N= 143) | Obese (N=78) | **p-value** |
| --- | --- | --- | --- |
| **Variable** |  |  |  |
| **Age** |  |  |  |
| Median (range) | 48.35 (26.5 - 78.8) | 51.78 (24.46 - 76.21) | 0.607* |
| **Stages** |  |  | 0.024** |
| I or II | 81 (58.3%) | 33 (42.3%) |  |
| III | 58 (41.7%) | 45 (57.7%) |  |
| NA | N=4 |  |  |

* Kruskal-Wallis test

** Pearson's Chi-squared test

Supplementary Table 3. Recurrence characteristics (n=28).

| **Type of recurrence** | **n (% from total)** |
| --- | --- |
| All | 28 (11.6%) |
| Local/First Event   - Ipsilateral - Contralateral | 6 (2.5%)  4 (1.7%)  2 (0.8%) |
| Distant/First Event   - Brain Metastasis   (Isolated Brain Metastasis)   - Bone Metastasis - Lung Metastasis - Lymphangitis - Liver Metastasis | 22 (9.1%)  13 (5.4%)  11 (4.6%)  3 (1.2%)  2 (0.8%)  2 (0.8%)  1 (0.4%) |
| Deaths   - BC-related death - Other Cancer - Cardiovascular - Infection | 20 (8.3%)  16 (6.6%)  2 (0.8%)  1 (0.4%)  1 (0.4%) |

BC: Breast cancer, pCR: pathologic complete response


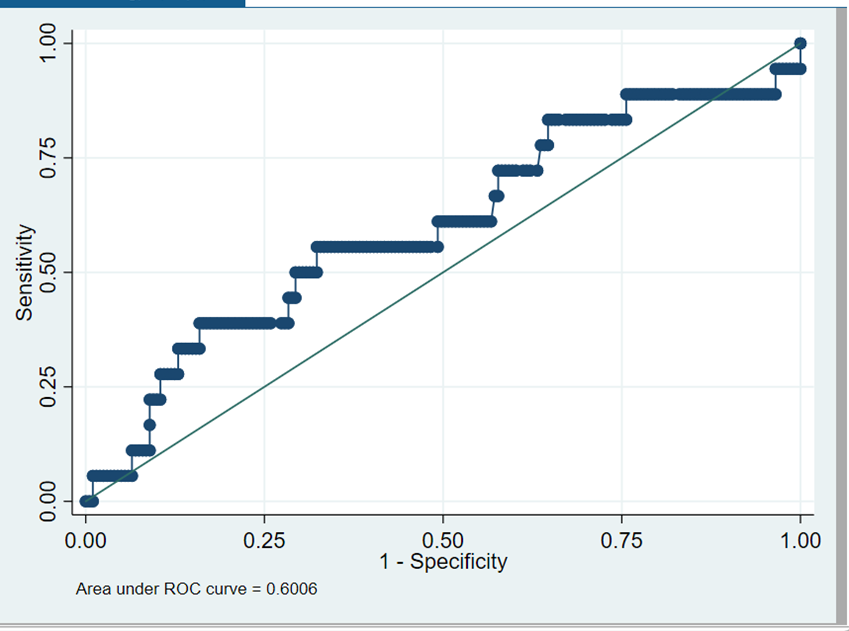


Supplementary figure 2. ROC curve of BMI versus patient OS
